# Biosensors for Monitoring, Detecting, and Tracking Dissemination of Poultry-Borne Bacterial Pathogens Along the Poultry Value Chain: A Review

**DOI:** 10.3390/ani14213138

**Published:** 2024-11-01

**Authors:** Nitish Boodhoo, Janan Shoja Doost, Shayan Sharif

**Affiliations:** Department of Pathobiology, Ontario Veterinary College, University of Guelph, Guelph, ON N1G 2W1, Canada; jshojado@uoguelph.ca (J.S.D.); shayan@uoguelph.ca (S.S.)

**Keywords:** biosensors, poultry-borne pathogens, antimicrobial resistance (AMR), on-site pathogen detection, sustainable poultry production, food safety

## Abstract

The poultry industry plays a crucial role in global food production, with chickens being the most widely consumed as a rich protein source. However, infectious diseases pose significant threats to poultry health, underscoring the need for rapid and accurate detection to enable timely intervention. In recent years, biosensors have emerged as essential tools to facilitate routine surveillance on poultry farms and rapid screening at slaughterhouses. These devices provide producers and veterinarians with timely information, thereby promoting proactive disease management. Biosensors have been miniaturized, and portable platforms allow for on-site testing, thereby enhancing biosecurity measures and bolstering disease surveillance networks throughout the poultry supply chain. Consequently, biosensors represent a transformative advancement in poultry disease management, offering rapid and precise detection capabilities that are vital for safeguarding poultry health and ensuring sustainable production systems. This section offers an overview of biosensors and their applications in detecting poultry diseases, with a particular emphasis on enteric pathogens.

## Simple Summary

The poultry industry is leading innovative livestock farming efforts by developing tools to monitor poultry gut health. Biosensors, as a groundbreaking technology, enable the rapid, on-site detection of bacterial enteric pathogens in poultry and poultry products across the value chain, addressing critical concerns like feed safety and antimicrobial resistance (AMR) monitoring. This review also examined specialized biosensor applications including in ovo pathogen detection and antimicrobial residue monitoring, which are pivotal for proactive health management. Furthermore, emerging innovations such as CRISPR-based biosensors are discussed for their potential to redefine pathogen detection capabilities. The review underscores both the technical strengths and practical limitations of these biosensor technologies, assessing their impact on enteric disease control, food safety, and sustainable poultry health management practices.

## 1. Introduction

The poultry industry is a cornerstone of global food production, providing a readily available source of protein through meat and eggs [1]. The demand for poultry meat and egg production continues to outpace that of other meats in the global meat market [2]. However, the industry faces significant challenges from infectious diseases that can cause severe economic losses and, in some cases, pose zoonotic risks to humans [3,4]. As researchers and industry professionals continue to grapple with the challenges faced by the sector, raising awareness of strategies available for improving disease detection and prevention in the poultry value chain will provide valuable insights for scientists, poultry producers, industry partners, and food safety experts. On this note, tackling the threat posed by zoonotic pathogens in the farm-to-fork paradigm is paramount. Avian intestinal pathogens such as *Salmonella* spp. (*S. enterica* serovars Typhimurium, Enteritidis, Inifantis, Virchow, and Hadar), *Escherichia coli* O157 and non-O157, *Clostridium perfringens*, and *Campylobacter* can spread rapidly through poultry flocks, leading to high mortality rates and reduced productivity. Subclinical infection can cause the contamination of poultry meat and eggs, leading to potential human infection. Human disease outbreaks linked to contaminated poultry meat and eggs are common globally, with *Salmonella* spp., *E. coli*, *Campylobacter*, and *Listeria monocytogenes* posing a particular concern in ready-to-eat processed meats [5,6,7,8] The CDC reports yearly outbreaks and detections of *Salmonella*, *E. coli*, *Campylobacter*, *C. perfringens*, and *L. monocytogenes*, with the first four being the top bacteria associated with foodborne diseases. Faster outbreak response and root cause identification are vital to prevent future disease occurrences in both poultry and humans [9].

The identification of pathogens that are the root cause of foodborne diseases requires rapid and smart sensing systems that can be deployed at any point in the poultry value chain. Over the last 20 years, smart poultry farming technologies have emerged, integrating sensors to monitor environmental parameters and poultry health [10]. Smart sensors, like biosensors, enable continuous real-time monitoring of flock health by tracking phenotypic and intestinal biomarkers such as core body temperature, activity levels, and vocalization [10]. These sensors can also be used to monitor and detect environmental conditions and biomarkers indicative of infection with pathogens such as *Salmonella* spp., *E. coli*, *C. perfringens*, and *Campylobacter* [11,12].

This review explores the role of biosensor technology in poultry disease management. This review was conducted in PubMed using a combination of specific keywords (chickens, diseases, detection, biosensors, in ovo, hatcheries, broilers, layers, market please, intestinal health, microbiome) as a search strategy. The resulting references were independently screened and relevant study methods, particularly focusing on the use of a biosensor, were extracted and used for this review. By focusing on recent advances in biosensor technology, this review aimed to examine the ways in which these technologies could be integrated into smart farming practices to monitor, detect, and prevent pathogen outbreaks in poultry flocks. The review will also explore the future potential of biosensors in supporting sustainable and safer poultry production practices by enabling earlier detection of infections and facilitating faster responses. Ultimately, this paper seeks to highlight the transformative role that biosensors can play in addressing the significant disease-related challenges faced by the poultry industry while supporting sustainable production practices.

## 2. Overview of Biosensors

**Figure 1:**
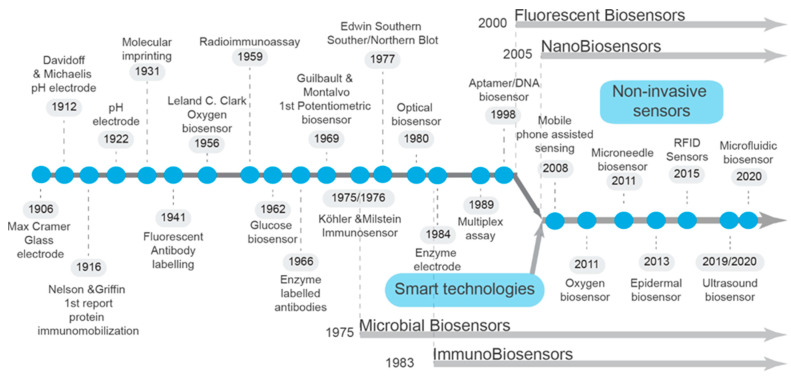
Historical milestones and a brief history of sensor technology and biosensors.

Biosensors are analytical devices that combine transducers and bioreceptors to detect pathogens through biological recognition elements (Figure 1). Bioreceptors capture targets using immunological (monoclonal antibodies), nucleic acid-based (complementary RNA or DNA), or label-free (glycan, lectin, or enzyme) methods and convert biological reactions into measurable signals by specifically identifying biomolecules within analytes [13,14]. Improving the robustness of biosensors for bench-to-field translation is essential due to the complexity of biological samples, which often include analytes from throat and airway swabs, fecal content, or blood components. Selecting appropriate bioreceptors is crucial, as their specific biochemical properties ensure high sensitivity and specificity, minimizing false positives or negatives [15].

The schematic representation illustrates the chronological advancement of biosensor technology, tracing its origins to Max Cramer’s invention of the glass electrode in 1906 [11]. This foundational development marked the beginning of a century-long progression in biosensor innovation. In 1922, Davidoff and Michaelis introduced the first hydrogen ion electrode, followed by Leland Clark Jr.’s groundbreaking creation of the oxygen electrode in 1956, which revolutionized electrochemical oxygen biosensing [16]. A pivotal moment came in 1959 with Yalow and Berson’s pioneering work on radioimmunoassays, utilizing I131-labeled insulin [17]. Biosensors gained traction in the 1960s. Guilbault and Montalvo introduced the first potentiometric enzyme electrode for urea detection in 1969 [18], while Divies proposed the first microbial biosensor in 1975 [19,20]. Concurrently, Edwin Southern’s development of DNA transfer onto nitrocellulose membranes in 1975 revolutionized DNA visualization, laying the groundwork for nucleic acid-based biosensors and aptamers [21]. Significant innovations continued into the 1980s including Lubbers and Oppitz’s fiber-optic sensor development and Kary Mullis’ invention of the polymerase chain reaction (PCR) in 1985 [22,23]. Liedberg and colleagues introduced the immunosensor in 1983, marking a milestone in biosensor evolution [24]. Subsequent decades have seen the integration of biosensors with nanotechnology, leveraging materials like silicon nanowires and gold nanoparticles for label-free or amplified sensing capabilities [11]. Today, biosensors have evolved into sophisticated devices incorporating smart technologies, enabling the non-invasive detection of physiological biomarkers and biological elements. These advancements underscore the transformative impact of biosensors across diverse fields, from medical diagnostics to environmental monitoring and agricultural applications.

Transducers convert these biochemical signals into measurable outputs via electrochemical, optical, or piezoelectric reporting units. Electrochemical biosensors detect changes in charge distribution on the transducer surface using potentiometric, amperometric, or impedimetric principles [25,26]. Optical biosensors, known for their versatility, enable multiplex detection against a single analyte by measuring optical signals following an interaction between the analyte and the recognition element. Piezoelectric transducers measure resonance frequency changes due to mass shifts when an analyte binds to the biological material, producing an electrical signal that can be analyzed to detect targets.

With advances in nanobiology and nanotechnology, the development of various biosensors for on-site utilization is becoming more prominent [27]. Accurate and portable, these inexpensive devices have the potential to be modified and applied toward the early detection of various pathogens in undefined asymptomatic conditions, or during disease outbreak, with high reliability and low cost. In the poultry industry, rapid detection and subsequent deployment of countermeasures can make the difference between survival or death and successful or poor performance, thereby substantially impacting sustainability and economic returns.

### 2.1. Principles for Application of Biosensors

**Figure 2:**
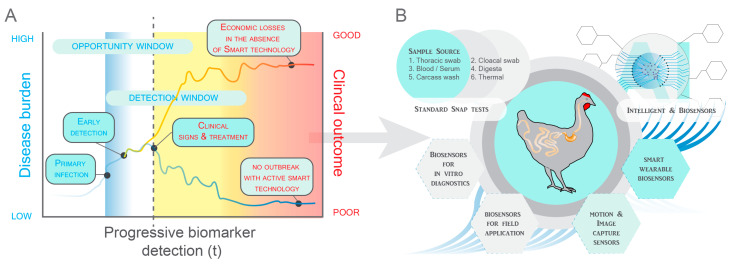
The early detection problem.

The seminal work of Clark in 1956 with the oxygen biosensor and Divies in 1975 with the microbial detection assay laid the groundwork for biosensors capable of early microbial biomarker detection through specific recognition elements [16,19]. Being able to define target elements and couple that with the rapid detection of specific microbial biomarkers paved the way for identifying specific diseases. Early disease detection is critical, especially when late-stage conditions complicate effective treatment or management, often resulting in significant morbidity and mortality (Figure 2). Detecting disease early also enables the containment of small outbreaks, minimizing adverse effects on surrounding environments.

The rapid detection of diseases in poultry is critical for timely diagnosis and treatment, reducing the risk of horizontal transmission and associated economic losses. A schematic overview of early disease detection strategies in poultry that integrated biosensors for detecting various biological elements or biomarkers is presented. (A) The challenge of early detection stems from the scarcity of biological elements or biomarkers indicative of disease onset. Currently, the main bottleneck in disease monitoring is the discrepancy between identifying infection by virulent pathogens and correlating it with specific stages of disease progression to justify prophylactic treatment. The concept of the detection window refers to a period during disease progression when the concentration of a biological element surpasses a sensor’s limit of detection (LOD), enabling its detection. In an ideal early detection system, this window should coincide with the early onset of disease and the opportunity window, which denotes the period during which intervention can effectively prevent disease progression. This opportunity window focuses on active screening rather than disease diagnosis. Progressive biomarkers are utilized to monitor disease progression from early stages to advanced phases. Effective early detection requires these biomarkers to surpass the detection threshold during the initial disease stages and within the detection window. In certain cases, the concentration of these progressive biological elements increases with disease severity over time. Following treatment application, these biomarkers may decrease below the LOD. If the detection window fails to overlap with the opportunity window, progressive biomarkers cannot expedite the implementation of early interventions. (B) The choice of sample source is pivotal for early disease detection. The source dictates when a biosensor can be deployed. Hence, biosensors can be used to target progressive markers during the detection and/or opportunity windows. Systems facilitating rapid disease detection generate data that support decision-making systems predicting the likelihood of disease outbreaks in poultry barns. The integration of smart technologies, such as motion capture sensors and artificial intelligence (AI), aids in early disease detection by monitoring activity levels and temperature changes in barns or individual birds.

There are two principal pathways for detecting microorganisms and diagnosing diseases: (1) active screening during asymptomatic periods based on risk factors, and (2) diagnosis based on observable signs. Application of an early intervention program can preempt disease outbreaks in poultry barns, as disease progression involves changes in its state once established. Early-stage disease spans the period from disease onset to the initial manifestation of signs. In most cases, following exposure and entry of an infectious pathogen into a susceptible host, replication and proliferation is initiated. While a resistant host may have short-term sub-clinical infection and eventually clear the pathogen, in a susceptible host, pathogen replication will often lead to overt clinical signs. The primary modes of infection, or early-stage disease, are via inhalation, skin contact leading to entry via an orifice, or the ingestion of contaminated substances. An established disease describes the period when clear signs of infection appear in a barn and treatments, or other actions are necessary.

In poultry barns, biosensors have significant implications for disrupting the “chain of infection”, which refers to the spread of an infectious agent into a barn from birds or humans through several linked steps, often facilitated by poor biosecurity measures or favorable climatic conditions (Table 1). Both active and passive surveillance programs typically rely on multiple reporting layers to inform specific actions. For example, monitoring infection in sentinel migratory animal species within defined breeding or wintering ranges can indicate potential future disease hotspots. These hotspots can then guide public health officials in identifying high-risk geographic areas for susceptible hosts. However, many infections remain undiagnosed due to inadequate diagnostic tools, leading to ongoing transmission and the delayed identification of emerging threats, with major economic consequences.

With the development of integrated systems and Internet of Things (IoT), continuous monitoring devices are expected to become more affordable. The data generated from integrated livestock monitoring can help poultry farmers improve animal welfare, health, and productivity. Therefore, there is a pressing need for point-of-care (POC) diagnostics and automatic, reliable detection as well as quantification tools that can predict disease occurrence before clinical signs appear in poultry.

### 2.2. Types of Biosensors

Biosensors have demonstrated considerable potential for disease detection in chickens, offering several platforms tailored to different detection needs including but not limited to immunological, nucleic acid-based, label-free, and wearable sensors (Figure 2). Antibody, aptamer, and nucleic acid-based biosensors can be combined with electrochemical, electrical, optical, or photoelectrochemical detection methods.

#### 2.2.1. Immunological Biosensors

Since the discovery of the antigen–antibody complex formation by Michael Heidelberger and Oswald Avery in the 1920s, numerous technical tools have been developed based on antigen–antibody affinity [28]. Today, this principle is advancing a new frontier in biosensors. Immunological biosensors are designed by immobilizing specific antibodies on a sensor’s surface to target pathogens of interest. When a sample containing the target biomarker or pathogen is introduced, the binding between the antibody and antigen generates a quantifiable signal that correlates with the pathogen’s concentration.

These biosensors are crucial in detecting infectious agents in poultry, leveraging the specificity of antibodies to recognize and bind to intracellular or surface-expressed antigens. Antibodies are particularly effective at recognizing proteins or complex lipid molecules, making them ideal for pathogen detection during infections. Currently, most sensors use polyclonal antibodies for detecting infectious bacteria [29]. The development of monoclonal antibodies are less feasible as it requires extensive screening for affinity and lack of non-specific binding.

In addition to antibodies, biosensors also employ nucleic acids and various enzymes, expanding the versatility and application or scope of these technologies in poultry health management. These advancements underscore the ongoing evolution of biosensor technologies, enhancing the efficiency and effectiveness of poultry disease detection and management.

#### 2.2.2. Nucleic Acid-Based Biosensors

Nucleic acid-based biosensors are a valuable tool for detecting the genetic material of pathogens during infection in chickens [30]. These biosensors utilize nucleic acid probes designed to complement specific regions of a pathogen’s DNA or RNA. When the target sequence(s) in a sample is(are) encountered, the probes hybridize with immobilized complementary sequences within the sensor, generating a detectable signal [31,32]. In chickens, these biosensors have been successfully used to detect pathogens such as *Campylobacter*, *C. perfringens*, *Salmonella*, *E. coli*, *Mycoplasma gallisepticum*, and *Campylobacter* [33,34,35].

A key advantage of using nucleic acid-based biosensors including aptamers in field settings is their stability under ambient conditions, unlike antibodies, which require more stringent storage and transportation. This stability is crucial for effective deployment in various environmental conditions. Additionally, nucleic acid-based biosensors excel at detecting pathogens during the early stages of disease onset or after transmission to susceptible hosts. Their ability to identify specific genetic sequences directly correlates with pathogen presence, enabling prompt and accurate diagnosis. As a result, nucleic acid-based biosensors can play a role in poultry health management, allowing for timely intervention and disease control strategies that improve flock health and productivity.

#### 2.2.3. Label-Free Biosensors

Label-free biosensors represent a significant advancement in biosensing technology by eliminating the need for additional labeling steps, thereby simplifying the detection process. These biosensors detect physical changes, such as refractive index, impedance, or mass, resulting from analyte binding. A key advantage of label-free biosensors is their suitability for on-site testing. By removing the need for complex sample preparation, which is a requirement for immunological and nucleic acid-based sensors, and minimizing cross-contamination risks, these biosensors enhance the efficiency and reliability of diagnostics in poultry farming. Their ability to directly measure analyte interactions with sensing elements simplifies pathogen detection, enabling rapid and accurate disease diagnosis. As a result, label-free biosensors significantly advance poultry health management by supporting proactive disease monitoring and control strategies within the industry.

The development and integration of advanced biosensors in poultry farming is essential to track and improve biosecurity and hygiene levels within hatcheries as well as grow-out barns to maintain poultry health.

## 3. Screening for Pathogens and Antimicrobial Resistance in Hatcheries

The process of egg incubation and hatching is a delicate process in hatcheries because this is the time when an egg, the developing embryo, and newly hatched chicks are highly susceptible to both vertical and horizontal transmission events. To limit infection during the earliest process, which is egg incubation, an antibiotic egg wash is often applied. Although a standard practice, the regular usage of antibiotics to sterilize eggs has been associated with an increase in the spread of antimicrobial resistant (AMR) bacteria [36]. It stands to the point that in ovo detection of pathogens and AMR is crucial for early intervention in poultry health. It enables the timely identification of disease-causing agents, reducing the spread of pathogens and minimizing the use of antibiotics. Early detection also supports the development of targeted vaccines and treatments. Ultimately, it contributes to more sustainable and safer poultry production by reducing the risks of zoonotic transmission and AMR emergence.

### 3.1. In Ovo Pathogen Detection

Poultry hatcheries are central to the poultry production chain. The transmission of pathogens into newly hatched chicks or embryonated eggs can lead to infection or mucosal colonization. Infection of an embryonated egg can occur horizontally through eggshells during and after oviposition or vertically during egg development. In vertical transmission, pathogens like *Mycoplasma gallisepticum* (MG) and *Mycoplasma synoviae* (MS), which are highly resistant to antibiotic egg wash, can penetrate the egg, leading to contamination of the yolk, albumen, and membranes [37]. In contrast, horizontal transmission following contact with fecal material, dust, or dirt can contribute to eggshell contamination. The contamination of eggs can occur via horizontal or vertical transmission due to the ubiquitous nature of *Campylobacter* and *Salmonella* [38,39].

The effective sterilization of eggshells by antibiotic dipping eggs can significantly reduce surface contamination. However, micro cracks within the eggshell can allow bacteria to penetrate, thus reducing the effectiveness of an antibiotic egg wash. Gram-negative bacteria like *E. coli* and *S.* serovar, which effectively penetrate the eggshell and infect the embryo, are often susceptible to antibiotics but resistant to the antibacterial components present within the albumen [40,41,42]. The subsequent infection of the embryo with *E. coli*, *S.* serovar Enteritidis, and *S.* serovar Typhimurium can affect embryo development without affecting its viability. Various studies have demonstrated that pathogens like *E. coli* and *S.* serovar, as detected in grow-out barns, can originate from hatcheries [40,43]. Therefore, in ovo detection of such pathogens is essential to prevent their subsequent dissemination into grow-out barns [42]. To break the chain of contamination between breeders and growers, biosensors can be applied to facilitate any decision-making process involving the removal and disposal of contaminated eggs from incubation. A wireless magnetoelastic (ME) biosensor was used, which allowed for the rapid, sensitive, and direct detection of *S. enterica* serovars Typhimurium on eggshells [44]. The proposed ME biosensor is a mass-sensitive biosensor that sends data via wireless signals to a define app. With this setup, the sensor is highly sensitive and able to detect *S. enterica* serovars Typhimurium on eggshells at concentrations ranging from 0.6 to 1.6 × 10^7^ CFU/cm^2^ within 30 min [44]. This new sensor might be more accurate and sensitive compared to polyclonal-based sensors considering the diversity of *Salmonella* serovars potentially generating false positive results [45]. Although *Salmonella* and *E. coli* represent the main zoonotic pathogens detected in eggs, the presence of *Campylobacter* contamination on eggshells has previously been observed. Infection is very rare and is not the main route for transmission. Therefore, such a sensor should be highly sensitive. Nucleic acid-based sensors are highly promising for the detection of *Campylobacter* in eggs as low as 1 CFU/mL [46].

### 3.2. Hatchlings

Post incubation, chicks hatched in hatcheries can encounter fomites on various equipment as secreted by infected chicks during processing, leading to their subsequent infection [37]. In fact, the presence of fomites contaminated with *S.* serovars or *E. coli* have been observed in fecal samples of newly hatched chicks [40,41,42]. This demonstrates the likelihood for the presence of *S.* serovars or *E. coli* within hatcheries or egg laying farms. Affected chicks of either layer of broiler genetics can present as weak with diarrhea and may die within the first week of life. Female chicks that survive are likely to be chronically infected with and consequently act as carriers of either *S.* serovars or *E. coli*. Hens with infection of the ovary may acts as vectors for further vertical transmission of either *S.* serovars or *E. coli* in eggs, leading to the contamination of hatcheries. In contrast, little is known about the consequence of subclinical infection in male chicks with *S.* serovars or *E. coli* that originate from hatcheries, Therefore, the process can be a man-made transmission cycle that is hard to disrupt, despite the best biosecurity practices.

Hatching chicks in house or in mixed farming systems can affect the newly hatched chick’s resistance to infection with *S.* serovars or *E. coli*. Chicks that are hatched within hatcheries tend to have an intestinal microbiome that is mainly populated by Gram-positive bacteria [47]. In contrast, chicks that are hatched in a mixed farming system with adult hens are more likely to acquire both Gram-positive and Gram-negative bacteria in the first day of life [48]. Acquiring a balanced microbiome within the first day of life can increase mucosal resistance and provide protection from early-life colonization by pathogenic bacteria such as *Clostridium* and *E. Coli*, especially during the grow-out phase.

Therefore, detecting pathogens in ovo offers an early intervention strategy that can prevent the spread of disease before hatching, thereby promoting healthier flocks. Unlike antibiotic screening or antimicrobial resistance, which addresses infections after they have occurred, in ovo detection helps reduce the reliance on antibiotics, minimizing resistance risks. This proactive approach supports sustainable poultry farming by enhancing biosecurity.

### 3.3. Antibiotic Screening

An essential component of livestock meat and egg targeted for human consumption is the need for appropriate antibiotic withdrawal time, so that products and by-products are antibiotic free [49,50,51]. Antibiotic residues such as tetracyclines, amoxicillin, quinolones, sulfonamides, β-lactams, cephalosporines, chloramphenicol, ciprofloxacin, and streptomycin are often traced in various poultry products. While antibiotics have been widely used in the prevention and treatment of infectious diseases, its sub-therapeutic usage to promote growth has led to improper use/abuse, leading to a surge in AMR [50]. In fact, significant levels of antibiotic residues have been traced in chicken meat, livers, giblets, and eggs [52,53,54,55,56,57]. This has led to various governments around the world implementing a ban and restriction on antibiotic usage in various livestock species and poultry. In 2022, the FDA released a report on antimicrobials sold for production animals, which stated that antibiotic sales per total weight of chickens have decreased by 70% since 2016 [58]. This antibiotic ban may not apply to the processes within hatcheries. Antibiotic usage during the process of egg dipping prior to incubation may well represent an animal health concern due to the risk of antibiotic resistance developing at the initial stage in the poultry value chain.

The most common method for identifying and confirming the presence of AMR genes (AMGs) is via polymerase chain reactions (PCRs) or metagenomics [59]. Metagenomics is an effective tool for whole microbiome sequencing and the discovery of new resistance genes. Both metagenomics and PCR methods require an extensive sample processing time before a result can be visualized. As such, alternative methods have been proposed. The most widely used technique for identifying antimicrobial susceptibility and the presence of AMG is the determination of minimum inhibitory concentration (MIC). MIC is the minimum concentration of antimicrobial that inhibits the apparent growth of bacteria in agar or broth medium. This method also lends to the identification or determination of a particular strain of bacteria that has acquired resistance based on a defined incubation time and antimicrobial concentration. Antimicrobial susceptibility testing against ampicillin has been applied to demonstrate the likelihood of resistance within *E. coli* strains obtained from clinical isolates [60]. Sun and colleagues proposed a colorimetric and electrochemical-based bioassay for the detection of *E. coli* using the p-benzoquinone as a redox mediator [61]. A visible color change captured with a smartphone could be used to identify the concentration of bacteria and demonstrate the presence of antibiotic-resistant bacteria [61]. A variety of label-free-based biosensors that utilize Raman and surface enhanced Raman spectroscopy (SERS) techniques have been developed for the identification of antibiotic-resistant *Salmonella* [62]. While many biosensors exist, few have focused on AMR, especially being able to demonstrate resistance to a particular antibiotic. The fast propagation of AMR and multidrug-resistant (MDR) bacteria is exemplified by the results of a recent Canadian study that showed a strong relationship between the pathogen *S. enterica* serovar Heidelberg and the commensal *E. coli* from retail chicken and human infections [63]. Once the biosensing component identifies AMR, the significance of it relies on further testing to determine which bacteria strains are present within the samples. The future for AMR testing would involve multiplex aptamer-based biosensors to demonstrate the presence of resistance markers as a more effective sensor for general application.

Beyond hatcheries, biosensors can be deployed for the detection of various contaminants, such as mycotoxins, bacterial toxins, and infectious pathogens, in real-time within feed used for grow-out barns.

## 4. Fungal and Bacterial Toxinotypes

To meet new standards for food safety regulations, significant effort is being invested to monitor the presence of various fungal and bacterial pathogens in feed or intestinal mucosa, respectively. In feed, the presence of fungal pathogens can cause digestive issues, while the presence of bacterial pathogens within the mucosa could be indicative of future disease outbreaks. Therefore, it is essential to track contaminants and additives within the scope of microbial food safety since they can integrate into the poultry value chain at any point in the process.

### 4.1. Monitoring Toxins

Toxin detection is a critical application for biosensors in poultry production. Intestinal pathogens including certain bacteria and fungi produce toxins that can negatively impact nutrient absorption. This is because these toxins can cause sloughing of the intestinal lining, leading to gastrointestinal disorders that compromise overall chicken health [64]. Additionally, chicken feed can become contaminated with toxins due to spoilage, and ingestion of these toxins can result in immunosuppression and secondary bacterial infections [65]. Therefore, the early detection of bacterial and fungal toxins is vital for implementing disease management protocols and preventing further complications within a poultry flock during the grow-out phase.

#### 4.1.1. Fungal Toxins

Mycotoxins are toxic secondary metabolites generated by *Aspergillus* species, *Penicillium*, and *Fusarium* species, which can be present in foodstuff and animal feed [66]. These toxins can have harmful effects including carcinogenic, mutagenic, immunotoxic, teratogenic, hepatotoxic, estrogenic, hemorrhagic, neurotoxic, dermatotoxic, and nephrotoxic impacts [67,68]. Major mycotoxins in animal feed include aflatoxins (AFs), ochratoxins (OTs), trichothecenes (such as T-2 toxin), and fumonisins (FBs), primarily produced by *Fusarium* species.

Eliminating mycotoxins or feed antigens from food materials is challenging. Managing mycotoxicosis in poultry requires the regular replacement of contaminated feed or bedding, as these toxins can spread within barn environments. Addressing subsequent immunosuppressive diseases is also essential, involving strategies like improved ventilation and the inclusion of organic acids, yeast, bacteria, and plant derivatives in feed. The use of antifungal agents further supports these mitigation efforts. Recently, phytobiotics have emerged as promising natural alternatives for sustainable farming due to their antibacterial, antifungal, antiparasitic, and immunostimulatory properties [65]. These can be applied on farms in bedding or feed and have potential as natural preservatives in poultry meat products, enhancing both food safety and sustainability in poultry production [65]. Beyond the farm, the bioaccumulation of mycotoxins in both broilers and layers can result in contamination of the meat or eggs.

Various biosensors have been developed to detect mycotoxins in feed, each offering unique advantages. Detecting mycotoxin-contaminated feed is crucial in poultry to mitigate the immunosuppressive effects of these toxic secondary metabolites [69,70,71]. Biosensors against both ochratoxin A (OTA) and aflatoxin B1 (AFB1) are essential due to concerns of OTA bioaccumulation and differential AFB toxin biotransformation between newly hatched chicks and that of adult chickens and between layers (female) and broilers (male) [72,73,74]. Sterigmatocystin (STEH), a precursor AFB1, is produced by fungal species such as *Aspergillus. flavus*, *A. parasiticus*, *A. toxicarius*, and *A. bombycis* [75]. Detection of STEH and AFB1 can be achieved using antibody-based and label-free smart biosensors [69,75,76]. Costa et al. demonstrated the selective detection of AFB1 as low as 0.79 pg/gram using a label-free electrochemical impedimetric immunosensor [77]. The sensor was developed using a gold electrode for the detection of AFB1 by a monoclonal antibody (anti-AFB1). In contrast, aptamers offer a cost-effective and highly specific alternative to antibodies. Cruz-Aguado JA and Penner G pioneered the development of a highly specific aptamer for detecting OTA [78]. Their work paved the way for the development of aptamers against AFB1, FB1, and T-2 toxin [79,80,81,82]. While aptamer kits are under development, more work is needed to optimize the manufacturing costs.

Lateral flow assays (LFAs) offer rapid detection and are increasingly valuable as point-of-care tools due to their short detection periods. Historically, LFAs detected only single mycotoxins, but advancements have led to multiplex LFAs capable of detecting up to six mycotoxins simultaneously [83,84,85]. These devices use different test line colors to indicate for the presence of specific toxins and their concentrations, which is supported by rapid semi-quantitative analysis via smartphone imaging.

#### 4.1.2. Detecting Bacterial Toxins and Antibacterial Responses

Biosensors are versatile tools to actively detect and monitor bacterial toxins and lytic enzymes, providing insights into the presence of or to actively track intestinal colonization, which could be essential for a predictive risk assessment. For example, assessing host antibacterial activity through changes in the lysozyme (LYS) enzyme offers an indirect method to gauge enteric infections [86]. In addition, quantifying ovotransferrin (OVT) expression in the cecum of broiler chickens also serves as an acute phase protein (APP) indicator for intestinal health [87]. By monitoring biomarkers such as APP or LYS, we can predict future disease or enteropathy based on changes within the ratio of intestinal Gram-negative and Gram-positive bacteria [88]. More specifically, changes in the ratio of intestinal Gram-negative and Gram-positive bacteria can be simply achieved by targeting 16S RNA sequences, thereby providing valuable insights into the intestinal microbiome composition [89]. In addition, understanding dynamic changes in the intestinal microbiota is crucial for differentiating between commensal and virulent bacterial strains [90]. This passive detection method, though non-specific, is crucial for identifying microbial shifts.

Endotoxins, or lipopolysaccharides (LPS), are components of the outer membrane of all Gram-negative bacteria such as *E. coli*, *Campylobacter*, and *Salmonella*. Detecting pyrogens like LPS and communicating their respective content levels is a matter of food safety concern. As a potent immunogen, LPS is recognized by immune system cells via Toll-like receptor 4 (TLR4), which induces the expression of pro-inflammatory cytokines [91]. The structure of LPS may vary among Gram-negative bacteria, leading to differing levels of binding affinity and subsequent inflammatory response induced via the TLR4 pathway. Toxicity, such as endotoxemia, only arise when LPS reaches the basal side of epithelial cells as Gram-negative bacteria penetrate through the mucous layer [91]. A biosensor utilizing polymyxin B was shown to be effective at detecting LPS from *E. coli* 0111 in the range of 0.2–0.8 ng/mL [92]. Additionally, an amine-terminated aptamer immobilized on a gold electrode was developed for LPS detection in complex samples, showing high affinity for LPS [93]. Additionally, Gram-negative pathogens such as Shiga toxin (Stx)-producing *E. coli*, *C. jejuni*, and *Shigella dysenteriae* pose risks to human and poultry health [94]. A multiplex lateral flow assay using an antimicrobial peptide has been proposed for the simultaneous detection of Stx in multiple microbes on a single strip.

In contrast, Gram-positive bacteria have a complex peptidoglycan exoskeleton, modified by compounds like teichoic acids and lipoteichoic acids (LTAs) [88,95]. The presence of LTAs within the gastrointestinal track can be sensed by TLR2, leading to the induction of inflammatory processes. LTA can be considered as a virulence factor that has an important role for infections by Gram-positive bacteria. Focusing on LTA does not lead to defining the presence of a particular pathogen, but it can provide basic information on changes within the intestinal microbiome or increased fermentation activity systemically, which could lead to health concerns. A point-of-care diagnostic assay has been created for detecting LTAs during bacteremia cases [96]. While various forms of LTA exist within *C. perfringens*, it is thought to play a key role in the infection and pathogenicity of various pathotypes and toxinotypes [97,98]. For instance, while *C. perfringens* strains commonly colonize the intestines of newly hatched chicks without causing immediate health issues, their proliferation can lead to immunosuppression or microbiome dysbiosis [99]. Various *C. perfringens* toxinotypes are responsible for a range of diseases in both humans and animals, ranging from subclinical disease to severe, life-threatening conditions [100]. Early toxin secretion may signal disease progression, while the detection of toxin-coding DNA can indicate early colonization by virulent pathotypes. Biosensors offer promising advancements in identifying and differentiating between various *C. perfringens* toxinotypes, which can speed up diagnosis. ELISA tests can detect elevated levels of alpha-toxin (CPA) in intestinal samples from chickens with necrotic enteritis (NE) and serum antibodies against CPA or NetB [101,102].

## 5. Monitoring Zoonotic Bacterial Pathogens in Grow-Out Barns

Common poultry-borne bacterial pathogens can be detected at various stages of food production: grow-out farms, processing plant, storage, and marketplace. *E. coli O157:H7*, *Salmonella spp.*, *C. perfringens*, *Shigella*, and *Campylobacter* are the major causative agents of foodborne diseases. Broiler chicks that originate from hatcheries and are positive for *E. coli O157:H7*, *S. enterica* serovar Typhymurium, *C. perfringens*, *C. Jejuni*, and *C. Coli* are more likely to transmit the pathogen to naïve chicks. While biosecurity measures are essential for controlling these pathogens, even the most stringent on-farm practices often fall short in preventing their environmental dissemination. In addition, poultry litter is an important experimental unit for monitoring the prevalence and emergence of pathogens before, during, and after grow-out periods.

Potential samples for detecting chicken-borne zoonotic bacteria at these stages include fecal samples, tracheal and skin swabs, and blood. Below, we discuss some of the major bacterial pathogens of chicken origin and highlight a few biosensors that have been developed that can be deployed for use in a barn setting.

### 5.1. E. coli

*E. coli* is a Gram-negative rod-shaped bacterium. Non-pathogenic *E. coli* is a naturally occurring intestinal bacteria in chickens, turkeys, and ducks as part of a healthy microflora [103]. Broiler chickens between the ages of 1 and 3 weeks are more susceptible to the pathogenic effects of *E. coli*, whereas layer chickens can be affected by pathogenic *E. coli* such as *E. coli* O157:H7 or avian pathogenic *E. coli* (APEC) throughout the growth and lay periods. APEC expresses various virulence factors such as toxins and transcriptional regulators, which contribute to disease pathogenesis in broilers. These virulence factors facilitate APEC colonization, the evasion of intestinal phagocytic cells, proliferation, and resistance to serum bactericidal activity [104,105]. The main challenge in *E. coli* diagnostics is distinguishing between pathogenic and non-pathogenic serotypes and strains. The development of *E. coli*-specific biosensors for application in chickens has focused on utilizing different recognition elements and transducer technologies to specifically detect pathogenic *E. coli* strains (O157:H7 and Stx-producing strains). A feasible way to distinguish between pathogenic and non-pathogenic *E. coli* of various serotypes is by using targeted single-stranded (ss)DNA aptamers against *E. coli* virulence factors. A lateral flow aptamer-based biosensor for the detection *E. coli* O157:H7 has been developed with a sensitivity level of 10 CFU/mL [34]. More recently, a gold nanoparticle-based aptamer was developed for the detection of Stx-producing *E. coli* [106]. As the *E. coli* binds to the aptamer, gold nanoparticles are released, and the concentration of *E. coli* can be determined by measuring the absorbance of free gold nanoparticles with a UV–Visible spectrophotometer. In this sensor, the detection limit was less than 1 log CFU/g, given that the running time for processing is less than 1 hour. For the detection of various *E. coli* strains and serotypes, monoclonal or polyclonal antibodies against surface/flagella antigens are available for application in immuno-based biosensors. For example, tests that utilize monoclonal antibodies or micro/nanobeads specific to *E. coli* O157:H7 antigens enable the detection of bacteria at concentrations ranging from 10 to 10^5^ CFU/mL [107,108]. In some cases, the detection limit has been shown to be as low as 10 CFU/mL for *E. coli* O157:H7 with an estimated detection time ranging from 30 to 120 min [107,109]. The performance and LOD of immunosensors can be improved from 10^6^ to 1.0 CFU/mL by enriching the samples through overnight bacteria culturing [110]. Jaffrezic-Renault et al. demonstrated that by utilizing magnetic nanoparticles coupled with anti-LPS antibodies, it was possible to enrich samples without the need for overnight cultures by capturing Gram-negative bacteria onto a graphite ink electrode [111]. Therefore, the use of immunomagnetic beads allows for the real-time purification of a sample from a complex matrix, leading to the sensitive detection of *E. coli* at 1–10^3^ CFU/mL. This is because, when detecting *E. coli* O157:H7 in various matrices, without an enriching step, it was discovered that the pathogens adhered differently to each of the matrices. Thus, an RCA-coupled DNAzyme amplification in an electrochemical assay yielded an LOD of 8 CFU/mL and a detection range of five orders of magnitude [112]. This assay highlighted a key challenge in distinguishing between the recognition of specific biological patterns and the non-specific adhesion of the microbe to a membrane. In contrast, a simpler stack-pad immunoassay that takes less than 5 min for detection has been explored for the detection of *E. coli* [113]. While the colorimetric stack pad immunoassay does not differentiate between pathogenic and non-pathogenic *E. coli* strains, it has the potential for rapid detection and demonstrates that it is possible to miniaturize a detector.

### 5.2. C. perfringens

In recent years, the broiler industry has seen an increase in incidence of infection with enteric pathogens like *C. perfringens* [114,115]. Primary infection with *C. perfringens* is via oral contact with contaminated fecal matter. In broilers, productive replication of virulent *C. perfringens* leads to the secretion of toxins that degrade the intestinal tissue lining, leading to NE [99,116,117,118,119,120]. NE manifests as ulcerative lesions along the small intestine and cecum [121,122]. The ability for the rapid detection of a microbe within a given detection window can reduce the need for antibiotic usage. Various *C. perfringens* toxinotypes (A, B, C, D, and E) expressing an array of different toxins (α, β, ϵ, and ι) are targets for the development of immunological biosensors. In fact, the detection of toxins can be suggestive of an active infection [100]. Immunosensors have been shown to be highly sensitive for the detection of the epsilon toxin as produced by virulent *C. perfringens* strains [123]. The sensor was developed by using an epsilon toxin-specific monoclonal antibody, with the sensor’s result comparable to that of an in vitro ELISA test kit against the epsilon toxin [123]. This sensor is based on a toxin-liposome disruption assay that is highly effective at demonstrating the binding of toxins to liposomes, leading to pore formation and subsequent detection of a fluorescent signal. Liposome-based sensors offer an avenue to determine whether the specific analyte contains other *C. perfringens* toxinotypes for which the anti-epsilon immunosensor is not specific [124]. Furthermore, it can provide insights into the functional role of a particular toxin. A more recent study demonstrated that membrane–protein interactions are an effective technique for the detection of the *C. perfringens* Perfringolysin O (PFO) pore-forming toxin, paving the path to a better understanding of membrane–protein interactions and the development of novel sensors that can detect these interactions [125]. While a combination of immunosensors or liposome can demonstrate the secretion of biologically active toxins, the limit of this technology is the fact that the sensor does not differentiate between the *C. perfringens* toxinotypes. The detection of DNA that encodes for toxins, where antibodies might lack specificity, can be used to identify and differentiate between virulent pathotypes or toxinotypes. A multi-toxin oligonucleotide microarray has been developed that can detect six different toxins produced by various *C. perfringens* toxinotypes [126]. Others have demonstrated the advantage of using oligoprobes that strongly hybridize to the corresponding toxin oligonucleotides [114,115,116]. After hybridization, the microarray is scanned to measure signals against the background as an indicator of bacterial presence in samples. This experimental approach has also been used for the simultaneous analysis of virulence genes from several bacterial strains on a single-chip platform [117]. In the case of *C. perfringens*, this type of rapid detection can help differentiate between virulent and avirulent pathotypes, which would be indicative of early life colonization.

### 5.3. Salmonella

*Salmonella* is a Gram-negative bacteria and a major zoonotic pathogen. In poultry barns, *Salmonella* asymptomatically colonizes the chicken intestine, serving as a reservoir for both vertical and horizontal transmission to naïve poultry. Infection with *S. enterica* serovar Pullorum and *S. enterica* serovar Gallinarum causes pullorum disease and fowl typhoid in poultry, respectively [127]. In contrast, *S. enterica* serovar Enteritidis and *S. enterica* serovar Typhimurium strains are associated with zoonotic infections transmitted through the consumption of contaminated poultry products [127]. The timely and accurate detection of *Salmonella* in chickens is essential for implementing effective control measures to prevent further disease outbreaks on farms. In contrast to antibody-based sensors, aptamers offer a highly sensitive method for detecting and differentiating between the various *S. enterica* serovars. Aptamer-mediated isothermal strand displacement amplification coupled with an LFA test was developed for the detection of *S. enterica* serovar Enteritidis [33]. The assay could detect *S. enterica* serovar Enteritidis at concentrations of 10 CFU/mL, allowing for semi-quantitative detection with a strip reader. In addition, Zhang and colleagues designed a novel capture probe composed of aptamers against a specific target sequence of *S. enterica* serovar Enteritidis [128]. An optical colorimetric biosensor used for the detection of *S. enterica* serovar Typhimurium ATCC 14028 using aptamer technology has also been developed with an LOD of 16 cfu/mL [129,130]. This type of biosensor is in its early developmental stages but has the potential for miniaturization, especially as it pertains to the detection of *S. enterica* serovar Typhimurium. While aptamer-based sensors can only define the presence of bacterial DNA, detecting bacteria cells can indicate intestinal colonization. Guo et al. developed a method for the specific detection of *S. enterica* serovar Typhimurium using catalase-modified antibodies that catalyze the conversion of hydrogen peroxide to water in the presence of *S. enterica* serovar Typhimurium, which results in a lower LOD of 35 CFU/mL [131]. These sensors have the potential to be utilized for the detection of various *S. enterica* serovars in litter.

### 5.4. Campylobacter

*C. jejuni* is a Gram-negative, commensal pathogen found in the intestinal tracts of a wide variety of agricultural animals including chickens. Avian species are recognized as a significant reservoir for *C. jejuni* [132]. In fact, *C. jejuni* has surpassed *Salmonella* as the major cause of food-borne bacterial diarrhea in developed and developing countries, a significant concern for public health [133]. Contaminated poultry products, such as raw chicken meat, can be a significant source of human infections [134]. As a result, in 2017, the European Commission established a criterion requiring frozen broiler carcasses to contain no more than 1000 CFU/g of *Campylobacter* bacteria [135]. Therefore, the rapid and accurate detection of *Campylobacter* within chickens is important to implement control measures and meet the regulatory standards. Sampling chicken droppings within a barn can provide an assessment for the presence of *C. jejuni* with a barn. A lateral flow test (LFT) based on anti–*Campylobacter* A and/or B allows for the testing of various barn samples as well as the cecal content in euthanized chickens. Using the LFT, it was confirmed that *C. jejuni* can be detected at an LOD of 6.7 log CFU/g in barn litter and 7.1 log CFU/g, respectively, within the cecal contents [136]. However, there are significant limitations associated with immune-based detection methods. Due to cross-reactivity between pathogenic and non-pathogenic strains as well as *C. coli*, some immunoassays may elicit false positives and undefined variability for clinical samples. The use of rabbit polyclonal antibodies to define the presence of *C. jejuni* on a biosensor developed with a surface plasma resonance (SPR) sensor platform has shown some early promise [137]. The sensor chips were assessed for an LOD ranging from 4.0 × 10^4^ to 8.0 × 10^6^ cfu/mL [137]. For lower LODs, the sensor should use monoclonal antibodies to reduce non-specific binding. Antibodies cannot distinguish between viable from dead cells. Furthermore, further tests will be required such as culture for the laboratory diagnosis of *Campylobacter* enteric infections. Another potential direction for the improvement of antibody-based biosensors would be to utilize monoclonal antibodies to identify ideal target epitopes to enhance the resolution of differentiating between different strains of *Campylobacter.*

## 6. CRISPR-Based Biosensors for Pathogen Detection

The clustered regularly interspaced short palindromic repeats (CRISPR) sequence was initially designed for editing a target host genome in cohort with the CRISPR-associated (Cas)9 protein [138]. These endonucleases are not only used for genome editing, but also have molecular diagnostic applications [139]. As powerful tools for nucleic acid sensing, modern biosensors based on CRISPR systems can be more widely applied for the detection and rapid diagnosis of zoonotic bacterial pathogens of chickens. Moreover, the CRISPR method can be performed at physiological temperatures, meaning that it is more feasible to assemble into POC devices. There are many CRISPR/Cas systems that are naturally available among the prokaryotes including Cas9, Cas12a, Cas13a, and Cas14a. Of these CRISPR/Cas proteins, Cas12a has gained significant popularity for usage in biosensors. Once activated, Cas12a can indiscriminately cut ssDNA, thereby facilitating the detection of specific ssDNA segments in a mixture.

### Cas12

Amongst these Cas proteins, Cas12a is a class 2, type V, RNA-guided DNA endonuclease, which is more versatile than Cas9 in editing genomic sequences and in molecular diagnostic applications. Cas12a-based biosensing was developed for detecting the genetic material of a variety of pathogenic microorganisms such as the *LMOSLCC2755_0090* gene of *L. monocytogenes* and invA gene of *S. enterica* [140,141]. The aforementioned *L. monocytogenes* biosensor enables highly specific detection, with LODs of 0.68 aM of genomic DNA and 940 CFU/g in spiked meat samples within 2 h [140]. In another study, a biosensor employing Cas12a for *S. enterica* successfully detected *Salmonella* genomic DNA at concentrations as low as 0.88 pg/μL and 1.28 pg/μL for fluorometric and colorimetric responses, respectively [142]. The specificity of the sensor was tested and demonstrated to lack cross-reactivity and specificity against various bacterial species including *Streptococcus pyogenes*, *Mycobacterium avium*, *E. coli* strain H10407, *Bacillus cereus* strain 971, *B. subtilis* strain 168, *C. jejuni*, *S. aureus* subsp. *aureus*, *Shigella flexneri* strain 24570, *C. perfringens*, *Aeromonas hydrophila* strain CDC 359-60, *Legionella pneumophila* subsp. *pneumophila* strain Philadelphia-1, and *Plesiomonas shigelloides* [142]. Zhu et al. showed the specificity of recombinase-aided amplification (RAA) and CRISPR/Cas12a technology for detecting *E. coli* O157:H7 [143], demonstrating a detection limit as low as ~1 CFU/mL (fluorescence method) and 1 × 10^2^ CFU/mL (lateral flow assay) with an assay run time of 55 min [143]. Most Cas12a sensors show reliability for the application and detection of bacterial targets in meats and more complex samples.

The intensification of poultry farming and the emphasis on improving slaughter characteristics, such as an increase in meatiness and a decrease in carcass fat content, can have negative consequences such as difficulties in maintaining meat quality. In addition, processing plants actively process samples from various farms and thus are a bottleneck site for pathogen accumulation. Due to these limitations, traditional methods such as carcass visualization are not sufficient to determine the meat quality and presence or absence of infectious pathogens. Therefore, higher emphasis is placed on defining meat quality at the processing plant before they reach the marketplace.

## 7. Processing Plant to the Table: Packaged Meat Quality Control

Nowadays, there is an immense need for biosensors in food processing operations to assess the product quality. Food product quality can be altered by environmental toxins, handling and processing, storage, and distribution, which can affect the physical, chemical, nutritional, and biological properties of food products [144]. According to a Food and Agriculture Organization report, 1.3 billion tons of food products are wasted yearly [145]. Therefore, knowing the environmental conditions that food products are exposed to within food supply chains is paramount, and various sensor technologies are being explored that can help provide these data. Various technologies have been developed to indicate packaging integrity issues and highlight meat spoilage during transportation from the processing plant to retailers [146].

### 7.1. Slaughterhouse Quality Control

During carcass processing in an slaughterhouse, poultry carcasses contaminated with *Campylobacter* or *Salmonella*, originating from infection at the farm level, or from in slaughterhouse cold wash can contaminate clean poultry products, leading to the major adulteration of larger batches of products [147,148,149,150,151]. Nucleic acid-based sensors are highly promising for the detection of the *Campylobacter* contamination of meat products. For example, a multiplex fluorescent assay was shown to be capable of differentiating between *C. coli* and *C. jejuni* in food samples [46]. The assay exhibited an LOD of 1 CFU per reaction as well as 1 CFU/mL and 10^3^ CFU/g when tested with analytes such as chicken broth and egg/meat samples, respectively. In addition, multiple DNA-based sensors were used for the detection of *Campylobacter* in poultry meat at an LOD of 1.5 × 10^1^ CFU/g [152]. A lateral-flow biosensor (LFB) with duplex loop-mediated isothermal amplification (d-LAMP) was demonstrated to be effective for the detection of *Campylobacter* spp. in experimentally spiked chicken meat samples [153]. The lowest inoculated detection limit for *Campylobacter* was 10^3^ CFU per 25 g of chicken meat samples.

### 7.2. Marketplace Quality Control

There is a lack of knowledge on how meat processing (freezing, cooking) can affect its nutritional properties and the development of unpleasant odors and aroma [154]. Tracking presence and the quantity of volatile organic compounds (VOCs) can provide an indication of a low quality or spoiled meat product. Rotting meat or degradation can lead to the production of VOCs. Maintaining low temperature is essential as it will restrict the growth of microorganisms involved in food spoilage [154]. Within the supply chain, biosensors can provide real-time information on the integrity of meat products by assessing temperature fluctuations, contact damage during transportation, and storage, which is a result of an improper or damaged packaging seal [155,156]. Damage to the seal can lead to the escape of antimicrobial gases, thereby leading to undesirable microbial growth as well as the production of volatile gases and liquids, making a product visually unappealing and unsafe for consumption [155,156]. For example, technologies like the Food Sentinel System provide alerts to consumers and retailers about the integrity of the product being purchased. In this smart biosensor, the presence of contaminated meat product results in the reporter generating a red color [157]. Similarly, Flex Alert (Vancouver, BC, Canada) and Toxin Guard™ (Toxin Alert Inc., Mississauga, ON, Canada) have developed commercially available biosensors that can detect toxins like *aflatoxins* and those produced by *E. coli O157*, *Campylobacter* spp., *Listeria* spp., and *Salmonella* spp. in packaged food [158].

The detection of *Salmonella* on various types of poultry packaging has been demonstrated using a microfluidic hand-held device [159]. The total assay time was 10 min and a detection limit of 10 CFU/mL was observed in all matrices, demonstrating the suitability of this microfluidic hand-held device for real-world use. Within the liver samples purchased from retailers, *C. jejuni* was found in off-the-shelf liver products, demonstrating the nature and persistence of this pathogen in poultry products [160]. Furthermore, an advanced genosensor, or aptamer based with silica nanoparticles, was shown to be able to detect the presence of *C. Jejuni* in chicken meat purchased from local markets [161]. Surface enhanced Raman scattering (SERS) was established for the ultra-sensitive detection and monitoring of *C. jejuni* in food [162]. This assay was highly effective for the detection of *C. jejuni* with an LOD ranging between 50 and 75 cfu/mL.

Alternatively, using a dual-mode sensing platform with a fluorescence and electrochemical aptasensor for the detection of *C. perfringens* alpha toxin DNA was demonstrated in artificially contaminated chicken and duck meat samples [163]. In this sensor, the electrode relied on an [Fe(CN)6]^3−/4−^ solution for electrochemical detection, while the fluorescence intensity was measured with a fluorescence spectrometer. The dual-mode sensor greatly improved the reliability of detection with an estimated limit of detection (LOD) of 1 CFU/g in both the artificially contaminated and market meat samples [163]. However, alpha toxin is not an essential virulence factor in NE disease progression, and biosensors developed for the detection of toxins such as NetB, which is crucial in the development of NE in poultry, would be more desirable [164].

The use of biosensors in predicting meat quality can be time saving (Table 2). Furthermore, the amount of research material needed is small and only requires the wash from meat samples as an analyte to determine the level of contamination. Therefore, biosensor technology can significantly improve meat quality assessment and reduce the cost of testing in meat plants and slaughterhouses. However, further work is required to develop types of sensors that are highly specific.

## 8. Conclusions

In conclusion, the integration of biosensors into smart poultry farming represents a transformative leap forward in managing intestinal diseases in chickens. By enabling early intervention, real-time monitoring, non-invasive sampling, high diagnostic specificity, biosensors offer a comprehensive approach to reducing antibiotic usage and improving flock health. Ideally, new point-of-care diagnostics for infectious diseases in veterinary settings, especially poultry, should adhere to the REASSURED criteria, ensuring that tests are Real-time, Easy to use, Affordable, Sensitive, Specific, User-friendly, Robust and Required, Equipment-free, and Delivered to those in need.

Innovative diagnostic tools that combine elements from existing laboratory-based tests, lateral flow assays, and emerging technologies such as synthetic biology and printed components hold potential for creating innovative diagnostic tools tailored to poultry health management while promoting disease prevention and responsible antimicrobial practices in the industry [165]. By enhancing food safety and contributing to public health improvements, biosensors are set to become a cornerstone of modern poultry production systems. The future of poultry farming hinges on continued research and development in biosensor technology, holding immense potential to enhance veterinary diagnostics, support sustainable poultry farming practices, and secure the industry’s long-term viability in an increasingly health-conscious global market.

## Figures and Tables

**Table 1:** Health and safety impacts for the implementation of biosensors.

Category	Key Point	Description and Pathogen Detection	Innovative Aspects	Strengths and Limitations	Example
Regulatory Compliance	Antibiotic residue detection in meat and eggs	Detects antibiotic residues, such as tetracyclines and sulfonamides, to ensure compliance with food safety regulations and protect consumer health.	Rapid, on-site biosensor application for screening residues in poultry products.	Strengths: Ensures product safety and regulatory compliance. Limitations: Sensitivity may vary for low residue levels.	Portable sensors detecting tetracycline residues on eggs before market distribution.
Feed Safety	Mycotoxin monitoring in poultry feed	Identifies mycotoxins (e.g., aflatoxins) in feed, preventing health risks like immunosuppression and secondary infections in poultry.	Field-based biosensors for on-site mycotoxin detection in feed supplies.	Strengths: Reduces mycotoxin exposure in poultry. Limitations: Validation needed for use across different feed types.	Aptamer-based biosensors for aflatoxin detection in poultry feed before distribution.
AMR Control Strategy	Impact on antimicrobial resistance (AMR)	Early detection reduces reliance on antibiotics, lowering the risk of AMR development and improving poultry health management.	Highlights biosensors’ role in reducing antibiotic use, supporting AMR control strategies.	Strengths: Minimizes antibiotic reliance, promoting flock health. Limitations: Initial costs may limit adoption.	On-site biosensors monitoring *Campylobacter* levels to inform non-antibiotic control strategies.
Pathogen Surveillance	In ovo detection in hatcheries	Detects pathogens (e.g., *Mycoplasma*, *Salmonella*, *E. coli*) in eggs, preventing transmission risks among newly hatched chicks.	Applies biosensors for in ovo pathogen detection, breaking the contamination cycle in hatcheries.	Strengths: Improves biosecurity, reduces disease spread. Limitations: Requires frequent calibration for accuracy.	Wireless biosensors detecting *Salmonella* contamination on eggshells in hatchery settings.

**Table 2:** Key technological advancements in biosensors for pathogen detection.

Category	Key Point	Description	Pathogen Detection	Novelty of the Work	Strengths	Limitations
Technological Advancements	Rapid, on-farm pathogen detection	Antibody-, Aptamer-, and label-free-based biosensors.	*Salmonella*, *Campylobacter*, *C. perfringens* *E. coli* *L. monocytogenes*	Routine and non-invasive monitoring across the poultry value chain.	Real-time, reduces economic losses and zoonotic risks.	Needs frequent calibration and testing for accuracy.
	Higher specificity for detecting and differentiating between pathogens	CRISPR-based biosensors for complex samples where traditional biosensors lack specificity.		Application of CRISPR technology in veterinary molecular diagnostics.	High sensitivity and specificity, thus minimizing cross-reactivity.	Requires some technical expertise for on farm use.
Pathogen Detection	Application in hatcheries (in ovo)	Prevent vertical transmission and limit antibiotic usage and limit the dissemination of AMR along the poultry value chain.	*Mycoplasma*, *Salmonella*, *Campylobacter*	Biosensor technology applied in ovo to break contamination cycles.	Improves hatchery biosecurity and chick health outcomes.	Specificity varies across pathogens.
	Post hatch (grow-out phase)	Simultaneously detects multiple pathogens across grow-out stages that pose high food safety risks.	*Salmonella*, *Campylobacter*, *C. perfringens* *E. coli*	Development of multiplex biosensors for on-site detection in poultry facilities.	Offers early detection, reducing spread and costs.	Cross-reactivity potential with commensal/non-target pathogens.
	Marketplace detection	Protects the consumer from foodborne illnesses during cold storage.	*Salmonella*, *Campylobacter*, *L. monocytogenes*	Early detection of food spoilage.	Builds consumer confidence in poultry meat.	Requires sampling every item being sold.

## Data Availability

No new data were created or analyzed in this study.
